# Participatory implementation research in the field of migrant health: Sustainable changes and ripple effects over time

**DOI:** 10.1111/hex.13034

**Published:** 2020-02-08

**Authors:** Maria E. T. C. van den Muijsenbergh, Joseph W. LeMaster, Parmida Shahiri, Michelle Brouwer, Mohammed Hussain, Chris Dowrick, Maria Papadakaki, Christos Lionis, Anne MacFarlane

**Affiliations:** ^1^ Department of Primary and Community Care Radboud University Medical Centre Nijmegen The Netherlands; ^2^ Centre of Expertise on Health Disparities Pharos Utrecht The Netherlands; ^3^ University of Kansas School of Medicine Kansas City KS USA; ^4^ Department of Health Services Research University of Liverpool Liverpool UK; ^5^ Graduate Entry Medical School and Health Research Institute University of Limerick Limerick Ireland; ^6^ Department of Social Work Hellenic Mediterranean University Heraklion Greece; ^7^ Clinic of Social and Family Medicine School of Medicine University of Crete Heraklion Greece

**Keywords:** implementation, migrant health, normalisation process theory, participatory learning and action methods, patient and public involvement, primary care

## Abstract

**Objective:**

This study aimed to explore whether positive impacts were sustained and unanticipated ripple effects had occurred four years after the implementation of interventions to improve cross‐cultural communication in primary care.

**Background:**

Sustaining the implementation of change using complex interventions is challenging. The EU‐funded “RESTORE” study implemented guidelines and training on cross‐cultural communication in five Primary Care sites in Europe, combining implementation theory (Normalisation Process Theory) with participatory methodology (participatory learning and action—PLA). There were positive impacts on knowledge, skills and clinical routines.

**Design, setting and participants:**

Four of the five original sites (England, Ireland, Greece, The Netherlands) were available for this qualitative follow‐up study. The study population (N = 44) was primary healthcare staff and migrants, most of whom had participated in RESTORE.

**Intervention; main outcome measures:**

PLA‐style focus groups and interviews explored routine practice during consultations with migrants. Etic cards based on the effects of RESTORE stimulated the discussion. Deductive framework analysis was performed in each country followed by comparative data analysis and synthesis.

**Results:**

Changes in knowledge, attitudes and behaviour with regard to consultations with migrants were sustained and migrants felt empowered by their participation in RESTORE. There were ongoing concerns about macro level factors, like the political climate and financial policies, negatively affecting migrant healthcare.

**Conclusion:**

There were sustained effects in clinical settings, and additional unanticipated positive ripple effects, due in part, from the participatory approach employed.

A short informative containing the major key words:

Implementation of change in primary care using complex interventions is challenging to sustain in routine practice.

Implementation theories can help identify levers and barriers to guide the development of action plans. Involving service users or community members can enhance analysis of levers, barriers and action plans.

An EU‐funded study, RESTORE, investigated and supported the implementation of guidelines and training initiatives on cross‐cultural communication in primary care, combined implementation theory (normalization process theory) with a participatory approach (participatory learning and action) and involved migrants along with other stakeholders in the implementation work. There were positive impacts on knowledge, skills and clinical routines.

This paper describes a follow‐up, descriptive study of the original RESTORE practices 3 years after the end of the original project. We found some qualitative evidence of sustained changes in clinical settings, and additional unanticipated positive ripple effects, as well as positive regard for the participatory approach employed.

## 1 INTRODUCTION

There are complex relationships between research, policy and practice in primary care, as well as increasing attention to translational gaps between them.^1^ To address these gaps, a number of strategies have been proposed for researchers. These include greater use of theoretical approaches in research focused on implementation^2^ and the use of participatory methods.^3^

Implementation science has grown rapidly in recent years, and a number of implementation theories are in use. Each offers a specific lens on the implementation process. For example, diffusion of innovation^4^ focuses on the introduction and spread of innovation in a clinical setting, while normalization process theory (NPT) has an extended focus from introduction of new practices through to embedding and sustaining them to the point that they are considered routine, that is normalized.^5^ Implementation theories, however, are rarely used prospectively, and it is not always clear how to operationalize them.^6^ Further, they are not designed to support individuals and groups through the development of action plans to shape implementation work in primary care.^7^ Participatory methodologies, on the other hand, do just that.^8^ All share a focus on including stakeholders affected by the issue under consideration and in a position to act on the findings to develop action plans (see http://www.ICPHR.org). Participatory health research and specific approaches such as participatory learning and action (PLA) can enhance public and patient involvement (PPI) and support implementation processes in primary care.^7^, ^9^, ^10^, ^11^, ^12^ There is also increasing recognition of the importance of the role patients/public may play in the broader, macro‐level context that shapes organizational capacity and willingness to take action to support implementation of changes in health‐care settings.^1^, ^6^, ^13^ In this paper, we provide results of a follow‐up study of an earlier investigation that combined NPT with PLA in primary care implementation research.

NPT is a contemporary sociological theory, developed by studying the implementation of health‐care innovations. It focuses on the work that stakeholders (eg clinicians, managers, patients) must do to introduce, integrate and embed a new way of working in daily routines until it is sustained in routine practice.^5^ NPT describes four elements of implementation work: *sense making, engagement, enactment* and *appraisal* (see Table 1). These have been used successfully as a conceptual framework to enhance understanding of levers and barriers to implementation for a variety of interventions.^14^, ^15^ There is, however, a lack of follow‐up studies in the literature exploring whether new routines have been sustained over time.^15^

**Table 1:** The four constructs of NPT (after Teunissen et al^12^)

Construct	What it addresses
Sense making	Can those involved in the implementation make sense of it?
Engagement	Do relevant participants ‘buy into’ the implementation work? Can those involved maintain their involvement and get others involved and engaged?
Enactment	What has to be done to make the intervention being implemented work in routine practice?
Appraisal	How can the intervention be monitored and evaluated? Can it be redesigned to sustain its use?

PLA is a practical approach to investigate problems among diverse stakeholder groups where asymmetries of power may exist.^16^ As such, it provides a valuable approach for meaningful, rather than tokenistic PPI in research. This is particularly the case with groups such as migrants who traditionally are underrepresented in PPI and in decision making in primary care.^17^ It has the capability to engage participants in a collegial, inclusive and active processes. This enables their authentic perspectives to emerge clearly in research outcomes.^18^, ^19^ The approach requires a specific *PLA mode of engagement*, which promotes values of reciprocity, mutual respect, co‐operation and dialogue within and across diverse stakeholder groups.^20^ *PLA techniques* (see Table 2) are inclusive and user‐friendly. These can be incorporated into interviews and focus groups in primary care research.^19^, ^21^, ^22^, ^23^

**Table 2:** Participatory learning and action (PLA) techniques (adapted from de Brún et al^7^ and O'Reilly‐de Brún et al^19^)

Flexible brainstorming	Fast and creative approach of using materials, such as pictures or objects, to generate information and ideas about the topic
Direct ranking	A transparent and democratic process that enables a group of participants to indicate priorities or preferences
Card sort	An interactive method for facilitating and recording brainstorming around topics. An emic card sort is based on ideas emerging from participants' knowledge and experiences. An etic card sort is based on a priori knowledge and experiences from, for example, previous research/discussions
Seasonal calendar	A grid‐based diagram used for co‐operative planning and democratic decision making. A flexible adaptive tool, it can be used as a ‘running record’ of stakeholders' planning over time
Speed evaluation	Short verbal or written evaluations, often used at the end of a PLA session to indicate (to participants and researchers alike) what key positive, negative and/or neutral experiences have occurred

The EU‐funded project RESTORE (REsearch into implementation STrategies to support patients of different ORigins and language background in a variety of European primary care settings; summarized in Table 3) combined NPT and PLA to investigate and support implementation of guidelines and training initiatives to improve communication in primary care with migrants who are not fluent in the language of their host country.^7^, ^11^, ^12^, ^24^, ^25^ There was evidence of positive changes to attitudes, knowledge and behaviour in practice settings^12^ (see Table 3).

**Table 3:** Information on RESTORE project (2011‐2015)

RESTORE was an EU‐funded qualitative case study project, which investigated and supported the implementation of guidelines and training initiatives that were designed to support communication between migrants and their primary care providers in five countries (Austria, England, Greece, Ireland and the Netherlands)^24^, ^25^
RESTORE was innovative in its combined use of PLA and NPT to guide methodology and provide a theoretical implementation framework.^24^ Throughout this process, the PLA approach facilitated health‐care providers to work collaboratively with migrants; to select and adapt a guideline or training initiative for their local setting; and to introduce it into their practice setting.^11^, ^12^ There were multiple impacts across settings. These included changes in knowledge (eg new knowledge and skills from completed training), attitudes (eg more tolerant and positive attitude towards migrant service users among receptionist staff) and behaviour (eg more effective communication in consultations between general practitioners and practice nurses and migrants with low literacy; increased flexibility in accommodating migrants' appointments among all staff). Impact on clinical practice routines was strongest in England and the Netherlands.^12^ Lack of resources for interpreting services in primary care and the impact of economic austerity reduced the impact in Ireland and Greece^12^

The use of PLA in RESTORE emerged as a key facilitator for the NPT implementation process.^7^, ^11^, ^12^ PLA enabled participants with different levels of knowledge and power—doctors, practice assistants and migrants—to work together in a democratic manner.^7^, ^11^, ^12^ This led to a shared feeling that the guidelines and training initiatives *made sense* (NPT construct 1), a shared sense of responsibility and *engagement* (NPT construct 2) to implement guidelines and training initiatives and shared work to *enact* them in daily practice (NPT construct 3) and *appraise* what difference they made, if any (NPT construct 4). It is not known, however, whether or to what extent these changes in knowledge, attitude and behaviour from the combined use of NPT and PLA, evident at the end of RESTORE, were *sustained* in practice over time (NPT construct 4). Furthermore, there is growing evidence that unanticipated benefits and ‘ripple effects’ may occur in participatory health research projects carried out in clinical settings, including primary care.^10^, ^13^ These are outcomes beyond the aims of the specific participatory research project and can include, as Bush et al describe, positive changes in relationships between service users and the empowerment of organizations' members.^13^ These matters have not yet been explored in relation to RESTORE and warrant investigation.

### 1.1 Aims and objectives

The aim of the present study was to describe the impact of the NPT‐ and PLA‐guided implementation of guidelines and training initiatives to improve cross‐cultural communication in primary care settings after a period of time.

Specific objectives were to:

1. Establish if changes in knowledge, attitude and behaviour introduced into daily routines at primary care sites, as part of the implemented guidelines and training initiatives in the RESTORE project, have continued;
2. Record migrants' and primary care staff's perceptions of reasons for the continuation or discontinuation of the changes in knowledge, attitude and behaviour; and
3. Explore if, and if so what, ripple effects in the primary care setting have occurred as a function of involvement in RESTORE.

## 2 METHODS

This is a descriptive, qualitative follow‐up study in four of the five primary care settings in Europe that were part of the RESTORE project and that were available for the follow‐up study—England, Greece, Ireland and the Netherlands. The fifth site, Austria, was not available for participation in this study. The RESTORE project ran from 2011 until 2015. This follow‐up study was performed during the summer of 2018. Ethical approval was obtained in all four countries.

### 2.1 Study population

The study population was primary care staff and migrants (community representatives working with NGOs/patients using primary care services) as well as other stakeholders (eg professional interpreters in Ireland) who participated in the general practices involved in the RESTORE project that were also involved in this study. At some sites, new staff members also participated. Participants for the follow‐up study in each country were recruited through purposive sampling, striving for representatives of each stakeholder group involved in RESTORE, via the RESTORE principal investigators (PIs) at each primary care site, using a combination of email and letters.

### 2.2 Data generation and analysis

Data were primarily generated using focus groups. Individual interviews were conducted to facilitate the involvement of those who could not attend the focus groups. Additional data were obtained based on the needs of participants: observation of clinical practice in the Netherlands because practice staff were unavailable for interviews, and email submission in Ireland for a participant who was abroad but wanted to take part. In England, given the interest of the local team, an analysis of minutes of meetings of a local policy group on cross‐cultural communication between spring 2015 and end 2018, as well as related documents, was conducted (see Table 4 data generation methods).

**Table 4:** Data generation encounters used in participating settings

	England	Greece	Ireland	The Netherlands
PLA style FGD N = 6	2 FG (12 participants)	2 FG (11 participants)	1 FG (5 participants	1 FG (4 participants)
Individual interviews N = 12	2		2 (by telephone)	7
‘Walking interview’ observation of practice				1
Observations of clinical encounters with migrant patients				3
Policy report analysis	Minutes of 8 meetings + other relevant documents; local policy organization on cross‐cultural communication			

A PLA etic card sort technique was designed for focus groups and interviews. This is an interactive method for facilitating and recording brainstorming around topics, which draws on relevant knowledge from previous research.^26^ For this study, 23 cards (see Table 5) were created, based on the impact of the RESTORE project on clinical practice^12^ which correspond to etic cards numbers 10, 11, 14‐16 and on examples of ripple effects from the participatory health research literature,^13^ which correspond to the other etic cards. The cards were used to stimulate discussion with participants about the objectives of this study: Have changes in knowledge, attitude or behaviour documented during the RESTORE project, continued or not? What are the perceived reasons for this? Are there any unanticipated ripple effects?

**Table 5:** PLA etic cards: possible ripple effects are shown in italic

Section 1. Changes relating to community participants	1. The reputation of the community organizations involved changed and affected other collaborations
	2. Community members' reputation beyond the organization and the project changed
	3. Community participants' awareness about specific diseases and stigma and taboo issues changed
	4. Community participants' sense of empowerment and confidence changed
	5. Community participants' appreciation of the value of research and evaluation changed
	6. Community participants gained new expertise which led to changes in other ethically sensitive research
	7. Community participants' assertiveness and confidence in venues in which they participated changed
	8. Community participants' willingness to take more risks in making suggestions, confronting issues, and encouraging and supporting others changed
	9. Community participants' influence in regional, national and international health‐care agendas changed
Section 2. Changes relating to clinical practice	10. Health‐care providers and staff used newly acquired research skills to work on service delivery for their community
	11. Participating clinicians' confidence in their health‐care consultations with migrants following training changed
	12. Clinicians' ability to think critically about and discuss their work openly changed
	13. Safety and patient‐centredness in participating practices changed
	14. Communication in consultations between migrants and clinicians changed
	15. Attitudes and tolerance towards migrants changed among clinical and administrative staff
	16. Migrants' confidence in the GPs' diagnosis and treatment changed
	17. Change in the primary care practice became apparent to other practices, who changed the way they engaged patients in their health‐care planning or delivery
Section 3. Changes relating to health research partnerships (relationships, interest in action research and new collaborations)	18. Relationships between the community, health care and researcher participants involved (in terms of mutual support and trust) changed
	19. Community, health care and researcher participants indicated changes in response towards action research methodology, and desire for more
	20. Led to new, related collaborations with other researchers and community groups
Section 4. Changes relating to academics	21. Academic members' community engagement in research in their academic circles changed (in amount, in approach)
	22. Researchers changed their research approach: their willingness to think about and share ideas with others and admit gaps changed
Section 5. Other data	23. Other spontaneously offered thoughts not related to any of the above topics

PLA focus groups were led by investigators from the original RESTORE project, who were trained in the use of PLA and co‐facilitated by medical students. The PLA focus groups were between 1 and 2.5 hours in length. Interviews performed by the students lasted approximately half an hour, in the language of the host country, as was the practice during RESTORE. They were audio‐recorded with consent of participants and transcribed for analysis.

Data analysis took place in pairs (RESTORE PI and student) in each country, in the language of the country, following the principles of deductive framework analysis.^27^ Specifically, the a priori categories for the deductive analysis were derived from the etic cards employed during data generation. These categories were used to develop a standardized template for data extraction, and coding took place using this template in each setting (see Appendix 1). Data were then translated into English (by the Dutch and Greek teams) to enable comparative analysis across settings.

A senior researcher experienced in qualitative research (JL) and a medical student (PS), who were not involved in the original RESTORE project, collated the standardized templates from all settings. Each of them independently examined the data recorded for the first etic category. Next, they compared and contrasted analytic notes to explore consistency of their independent coding. They then completed analysis of the remaining etic codes, asking three analytic questions based on the study objectives. They discussed emergent similarities and after discussion resolved any differences in their interpretation.

This analysis was discussed in three data analysis clinics by phone with the original RESTORE PIs of the four sites (CL&MP, CD, AM and MV) for further discussion and interpretation. This stepwise, critical analysis of the data enhanced quality and rigour of the interpretation of themes.

## 3 RESULTS

### 3.1 Study participants

There were 40 participants in this study who also participated in the RESTORE study (65% of the sample in the original study (N = 63). Other participants in the RESTORE study had moved away or were otherwise unavailable. In addition, four new practice members in the UK participated. Table 6 provides a breakdown per country and information on socio‐demographic and professional backgrounds as well as a comparison with the participants in the original study.

**Table 6:** Participants in 2019 follow‐up study: numbers in the original RESTORE project are shown in parentheses

Participant characteristics	England	Greece	Ireland	Netherlands
Total number	14^a^ (10)	11 (21)	8 (16)	11 (16)
(A) Gender				
(a1) Male	6	1	4	2
(a2) Female	8	10	4	9
(B) Age group				
(b1) 18‐30	2	3	1	2
(b2) 31‐55	7	8	7	9
(b3) 56+	5	0	0	0
(C) Country of origin/ ethnicity	England: 8 India: 2 Iran: 2 Pakistan: 2	Greece: 10 Iraq: 1	Ireland: 3 Congo: 1 Poland: 1 Russia: 1 Portugal: 1 Syria: 1	The Netherlands: 5 Turkey: 3 Morocco: 1 Syria: 1 Turkish‐Kurdistan: 1
D) Background/function				
Migrants (community representatives/care users	4 (5)	1 (2)	1 (5)	2 (3)
Primary care doctors	6 (2)	2 (4)	0 (2)	2 (2)
Primary care nurses	0 (0)	3 (5)	0 (0)	1 (3)
Primary care admin/management staff	2 (1)	2 (1)	1 (2)	5 (3)
Interpreting community	0 (0)	1 (0)	3 (3)	0 (1)
Health service planning and/or policy personnel	0 (0)	1 (7)	1 (1)	0 (1)
Researchers	2 (2)	1 (2)	2 (3)	1 (3)

a In England, the number of participants was more than originally in RESTORE because some GPs in the focus group had joined the practice more recently. In this results section we used some abbrevations which are explained in table 7.

**Table 7:** Legend to results section

In this section, the following abbreviations are used:
Eng = England
Gr = Greece
Ire = Ireland
Nl = the Netherlands
MIG = migrant (community representative or migrant care user)
PCD = primary care doctor (general practitioner)
PCN = primary care nurse
PCA = primary care administrator/management staff
IC = interpreting community
HSP = health service planning and/or policy personnel
Numbers in parentheses refer to the etic card that the result is based on

### 3.2 Continuation of changes in attitude, knowledge and behaviour

#### 3.2.1 Attitude and knowledge

Changes in attitude and knowledge from the RESTORE project continued in all settings. This primarily related to a more migrant‐friendly attitude and awareness about migrants' needs (Eng, Nl, Ire, Gre15).

There is more awareness, more awareness regarding this population. (Nl; PCD)

But I would say definitely attitudes toward migrants changed among clinical administrative staff.[….]it was a huge change between before and after RESTORE. […]they were very keen on understanding the words of migrants and very well informed about migrants' issues. (Ire; IC)

Yes … you can see this equal treatment […] It is clear when someone changes and treats you differently. (Gre; MIG)

In England, there was more awareness among practice staff about cultural differences; in the Netherlands, there was more awareness of the impact of low literacy on patients' understanding (Eng3; Nl13).

In both England and Greece, it was suggested that migrants had therefore become more confident in GPs' diagnosis and treatment (Eng,Gre16). In Greece, students and residents who were engaged in RESTORE had become more culturally sensitive professionals (Gre10). Their whole team had improved in practices towards migrants (Gre13).

Within the RESTORE period, I treated a Greek lady who wished to remove her intrauterine device but not […]by a man […]. We invested a lot of time to find a female gynaecologist and faced sarcasm by other professionals … they used to say ‘Put yourself together … this is Greece' … Now we don't have to find arguments to persuade the staff. (Gre;PCD)

However, some participants in Greece did not believe there had really been a change in attitude.

I think that people have not changed at all. To my view […]those people who were sensitive before are still sensitive. The rest have not changed. (Gre;PCA)

In the Netherlands, one of the receptionists expressed a negative sentiment against migrants.

Empathy and understanding is low in these patients. When they are late for their appointment they still want to be helped, saying something like: ‘last week I had to wait one hour, so now the doctor has to wait one hour for me’. (Nl;PCA)

#### 3.2.2 Behaviour—communication skills

Communication skills learned during the RESTORE trainings in England and the Netherlands and incorporated into daily routines were still being employed. In England, several PCD respondents reported greater confidence in communication during consultations with migrants and more patient‐centredness (Eng13). In the Netherlands, the health‐care professionals mentioned specifically that they now apply the so‐called ‘teach‐back' method^28^ to ensure what the patient had understood (Nl14).

During the [RESTORE] training I learned to ask if the patient want to repeat what I said. Often, they tell me a whole different story and it turns out that they did not understand what I was saying at all. (Nl;PCA)

#### 3.2.3 Organizational changes

There were reports of continued practical changes, including longer appointments and use of speaker phones to enable interpreted consultations in England (Eng14). Similarly, drawing on interview and observational data in the Netherlands, longer consultation slots were still planned in case of language differences. They had implemented and continued to use easy‐to‐understand patient information and pictograms, which improved greatly the understanding and accessibility of services for migrants.

Especially the pictograms had a big effect. It is much clearer for patients where they need to go, we do not have to point directions that often anymore. (Nl;PCA)

In Ireland, the practice manager explained that practitioners were more aware of migrants' issues, but there had been no change in the actual practice during consultations (Ire10,13,14). While the doctors knew that using a trained interpreter was the correct thing to do, they still did not have resources from the health service to do so. Staffs did, however, change behaviours at the reception desk and spent more time exploring ways to better support communication with migrants when they could:

Awareness, yes, absolutely, in the general sense […] that we understand where they're coming from a bit better, that there are better solutions to them than we can currently provide but ultimately we're still providing the same solution to the same problem to the same people.(Ire;PCA)

[We used to say] ‘Oh they’ve come for an appointment, they don't speak English, well we'll just have to figure it out.' Whereas now […]we might be more inclined to say ‘[…] let's put this appointment off for a week and see if we can figure out a way that we can communicate with this person'. Knowing that […] from all we've learned during RESTORE […]. (IrePCA)

### 3.3 Reasons for continuation or discontinuation of changes in knowledge, attitude and behaviour

#### 3.3.1 PLA enabled ongoing effect

The continued effect of the RESTORE was associated by many participants with the use of PLA to develop and implement action plans. Reflections on PLA included the following:

More the participatory aspect of it […]seems to lend itself to being a really good way of gathering information from the various strands. (Ire;PCA)

You don't feel as subject of research but as part of a friendly discussion. (Gre;PCD)

[…]I think I got a lot out of it [RESTORE], it was probably the best project and […]it was people who were all prepared to learn from each other […]. (Eng;MIG)

Although in the Netherlands, the doctor and nurse who had been involved in the PLA sessions during the RESTORE project were positive about the participatory approach, they were unsure about its long‐term influence. In fact, it seemed they remembered little about the PLA sessions and their participation (Nl19). The clinic manager also mentioned that the clinical staff felt totally overburdened by their current clinical load at the time of the follow‐up interview.

I have been through a lot these last couple of months, my head is very full. I cannot remember a lot of the things that were addressed during RESTORE. (Nl;PCA)

#### 3.3.2 Quality of the training

In the practices in England and the Netherlands, the continuation of RESTORE's effects was seen to be a result of the quality of the training programme. In line with the findings above, participants reflected positively on the participatory approach to co‐design training, that is using role play (Eng15), RESTORE's willingness to tackle difficult issues including racism, and the involvement of both administrative and clinical staff. It was also helped by the ongoing commitment of the participating practice to high‐quality health care for migrants (Eng14,15,16).

It [RESTORE training} was quite a few years ago but it's quite vivid. (Eng; PCD)

I think the communication training sessions created a bigger awareness of low literacy and low health skilled patients. We really changed it with the pictograms. (Nl;PCD)

#### 3.3.3 Lack of funding and local context limits further implementation

At the same time, at all sites contextual factors had a negative impact on the possibility to disseminate the results of RESTORE and implement them on a wider scale. National policies perceived to create a migrant‐unfriendly climate negatively influenced further implementation in England. In the Netherlands, lack of finances hindered dissemination; that is, further training of practitioners did not take place so overburdened primary care doctors and staffs did not use interpreter services. As well, there was a lack of reimbursement for the use of interpreter services. There was little evidence of the RESTORE training initiative being rolled out to other primary care teams in the cities where RESTORE took place in England or Greece. Proposals to increase migrant patients' access to statutory psychological services have also not been implemented in England (Eng17,18) or Greece.

I felt it was a really good project and I thought it had a wonderful effect on [the practice], the obvious problems of people not communicating and just a simple thing to show people that how to treat asylum seekers and refugees, I thought that was wonderful, almost magical […]

I've been terribly depressed to find that hasn't continued.

With resources it [RESTORE training] could have spread […] (Eng, different participants)

There was a strong view from one migrant that the broader political context, including Brexit and a hostile Home Office environment towards migrants, was likely to limit any changes resulting from RESTORE (Eng3,4).

In the middle of Brexit, things are probably worse than they were. (Eng;MIG)

In Greece, the changes in the participating primary care practice had been noticed by other practices, but overall the effect of the RESTORE project was felt to be limited to the participating health‐care centre, due to the limited spread of the results to the local community and other primary care settings (Gre9,17).

[…] This means that the effect of RESTORE was clear in the local context, where the pilots were carried out, while other settings […] did not gain sufficient connection with the project and its outputs. (Gre;PCA)

In general in Greece, participants were unsure to what extent changes in attitude were due to the RESTORE project itself, or the fact that more refugees had entered the country since RESTORE, as a result of which recent laws had improved accessibility to services for migrants and availability of interpreters (Gre2,9,7).

I don't really know if it was RESTORE or something else but I am sure that all these interventions engaging us in a dialogue on how to solve migrants' issues, helped us a lot in practice. (Gre;PCD)

It was emphasized, however, that despite the increase in interpreters' number during the past few years, they were still insufficient to meet the increased needs of the refugee population. Some other Greek respondents thought people had not changed at all (Gre3) and felt the whole health‐care system was overburdened due to the economic crisis (Gre7).

Furthermore, despite evidence of sustained, positive changes in the participating practices, participants explained that there was still scope for further improvement. In England, they had continued concerns around the use of interpretation services related to time delays during face‐to‐face consultations, interruptions to clinical communication when using telephone translation, and perceived problems with confidentiality when involving local community members (Eng14). Similarly, in the Netherlands and Ireland, participants described their continuing concerns about the poor availability of resources for interpretation services and the negative impact of family members or practice members acting as informal interpreters (Nl;Ire21). In the Netherlands, frequent changes in practice policies and staff hampered the ongoing effect of the interventions as well.

### 3.4 Ripple effects

#### 3.4.1 Community participants' empowerment

Although not to the same degree everywhere, migrant participants at all sites reported that they felt empowered by their participation in the RESTORE project, or by the attitude of the participating health‐care professionals. Some—but not all—of the migrants in the English setting considered that participation in RESTORE had enhanced their reputation, confidence and sense of empowerment.

We certainly passed on [a] few messages on RESTORE when we worked in another parts of the health service, that's the best impact it has had on us. (Eng;MIG)

A small charity being involved with a university professor and going into the [health authority] at a high level and sitting around a table and saying these are our findings, these are the improvements we like to see. (Eng;MIG)

Following their engagement with the university and their participation in international research and policy meetings, several participants felt their expertise is now better recognized and that they have greater impact, both locally and internationally.

I think the trustees were very pleased from a charity point of a view that we [Chinese well‐being organisation] were involved in this sort of project. (Eng;MIG)

But also:

I don't think so, because the hostile environment that people have talked about recently has had an overwhelming effect on people, and that would squash any other attempt to empower people. (Eng;MIG)

The migrant community participants in Ireland described the ways in which being in RESTORE, in an interstakeholder dialogue with more powerful figures (general practitioner, practice manager and policy planner), was a good experience for them. It empowered them in all aspects.

I think I had too much respect for authorities before and it hasn't made my life any easier or better […] you have to communicate these things. If you think that something is wrong it is really important to actually voice it […] going through this process and communicating with General Practitioners and policymakers kind of elevated my self‐respect and I try to deal with these issues better now. (Ire;IC)

An interpreter in Ireland explained that she was more assertive and direct with GPs about the importance of using trained interpreters, impacting their patient interactions:

[I say to GPs] ‘it is still your duty to inform the patient of everything he needs to know before he makes a decision’. A bit more polite than this but that is what I meant when I talk to them and before [RESTORE] I wasn't so confident. (Ire;IC2)

#### 3.4.2 New collaborations for research and policy

At all sites, the academic/researcher participants changed their research approach after the RESTORE project and were more able and willing to engage in new participatory research, to think about and share ideas with others and admit gaps. This led to new national and international initiatives and collaborations for policy and research (Eng,Gre,Ire,Nl20,21,22).

In England, the practice that participated in RESTORE is now recognized as an example of best practice for migrant primary health care in the city (Eng17). It may be best to consider its involvement in RESTORE as a catalyst for continued quality improvement. Community participants indicated that the RESTORE programme was part of a wider policy move towards social inclusion within the city in which RESTORE took place (Eng23). After RESTORE, two new local initiatives arose that have enabled policy and research initiatives to be extended across the city (Eng18,21).

In the Netherlands, the way the practice adapted to the needs of low‐literate patients was seen as an example for other practices:

Well the pictograms, which are very visual, are available for other practices and I know one practice also uses them now. So other practices are aware of this.(Nl;PCD)

As a spin‐off of RESTORE, some of the migrant participants in Ireland took part in new collaborative projects with the academic and policy planners—a national working group to implement trained interpreters in the Irish health‐care system (Ire18). They, as well as the primary care practice, became inspired to get involved in other participatory action research projects (Ire19,21).

[…] just from my perspective it was a fantastic learning opportunity, a fantastic opportunity to bring academia and the real world together. (Ire;PCN)

## 4 DISCUSSION

### 4.1 Main findings

There were examples of sustained changes in attitude, knowledge and behaviour in the four settings that were followed up four years after the implementation of the NPT‐ and PLA‐guided implementation of guidelines and training initiatives to improve cross‐cultural communication. This continuation was considered by participants to be due in part to the participatory methods used and the consequent involvement of all stakeholders in the development of action plans and training. Sustainability, however, was limited in several areas due to constraints in time and funding, especially for face‐to‐face interpreter services. Contextual factors were considered to be of influence as well. These were discussed as either hindering further implementation of good practices (eg as the result of the hostile political climate towards migrants in the UK) or promoting them (eg the larger‐scale implementation of migrant‐friendly services in Greece, due to the ongoing influx of migrants).

Besides these ongoing effects of the original, planned implementation work, ripple effects were also visible in the four sites, most notably the empowering effect that the participating migrants attributed to their experience of PLA methods. Also, some primary care practices now acted as examples for other practices and at all sites. The academics involved changed their behaviour towards new or more extensive policy‐oriented collaborations, and networks for migrant research and support have been developed.

### 4.2 Comparison with literature

Our results support the conclusion of the original RESTORE project that PLA is a key facilitator for supporting a sustained implementation process.^7^, ^11^, ^12^ The use of PLA emerged in participants' accounts across the four settings as an effective strategy to support the introduction of new ways of working in daily routines. Migrants and other participants developed relationships and collaboratively selected, adapted and introduced guidelines and training initiatives during the original RESTORE project. Our follow‐up study shows that the changes *continue to make sense* (NPT construct 1) to participants who see the value of health‐care adaptations for migrants and *remain engaged* (NPT construct 2) with new practices. This indeed highlights how the combination of NPT with PLA is a promising approach for investigating and supporting the implementation of complex interventions in daily practice to the point that they are considered routine, that is normalized.^5^

PLA also appears to be the main reason for the unforeseen positive ripple effects, like the empowerment of migrants and the changed attitude of researchers involved. This is in line with the review by Bush et al that revealed a range of positive yet unanticipated effects of participatory research projects.^13^ That review also underscored the need for all partners to agree on the importance of the research focus. Even more importantly, the likelihood of a participatory project exhibiting at least one extra benefit is quadrupled when the impetus for the study comes from a community organization.^13^ RESTORE was instigated by academics; thus, support for this community impetus would be a recommendation for further participatory research projects.

The influence of contextual factors, such as political climate, on the implementation and sustainability of changes in primary care was also found in the review by Lau et al,^1^ who postulated that the ‘fit’ between the intervention and the context is critical in determining the success of implementation. This is well documented in global health and Indigenous health in Australia and Canada.^29^, ^30^, ^31^, ^32^ Implementation research in the field of migrant health should consider these macro‐level influences on the process and outcomes. To achieve changes that are really sustainable, funding and manpower, a favourable political climate as well as the ambition to take new ways of working further is required.^33^

This highlights a key lesson learned regarding the value of having more senior‐level decision makers involved in participatory dialogues in primary care settings. Would the involvement of senior‐level decision makers improve the mobilization of resources after successful small‐scale pilots? Can such pilots projects generate change by reshaping policy agendas? These issues warrant further research.

### 4.3 Methodological strength and limitations

This follow‐up descriptive study is one of the few to assess the sustainability of the implementation of an intervention in primary care after four years. The involvement of new ‘external’ research team members not involved in the RESTORE project (JL, PS,MB,MH) and some new participants in England) helped to mitigate prejudiced conclusions that might have arisen from the sole involvement of the original RESTORE Investigators.

The study, however, has its flaws. One of the original RESTORE project countries is not represented, and the results might have been different if all countries participated. Also in question is whether theoretical saturation was reached, as it was not possible to contact all previous RESTORE participants: some had moved away and others were not available. The risk exists that those who did respond were more positive than non‐responders. The response rate, however, was reasonably high at 65%. The available self‐reported data were elicited during encounters that involved the original RESTORE PIs. This could have biased participants' responses positively, particularly given the relationships that were built during the original RESTORE project. Measures taken to address this during fieldwork included presentation of neutral statements about changes and specific probing about negative views. Participants did, indeed, report positive and negative views. Participating Dutch doctors found it difficult to recall precisely the RESTORE training, but this was not an issue at the other sites. As this was a complex intervention impacted by changing political contextual factors, it remains difficult to confidently attribute certain processes to the RESTORE intervention. Thus, we would be cautious about identifying direct cause and effect.

## 5 CONCLUSIONS

Implementation research in primary care that uses participatory approaches supports the introduction of new ways of working in routine practice that can be sustained over time. Further, the use of a participatory approach yields additional, unanticipated, positive effects on all participants. Participatory implementation research should be used to investigate and support other innovations for other populations in primary care.

## CONFLICT OF INTEREST

No conflict of interest.

## Data Availability

The data that support the findings of this study are available on request from the corresponding author. The data are not publicly available due to privacy or ethical restrictions.

## APPENDIX 1 CODING TEMPLATe

**Table:**

Topic/Data	Ireland	England	Netherlands	Greece
Any continued change in knowledge?				
Any continued change in attitude?				
Any continued change in behaviour?				
Migrant perceptions for continuation/discontinuation				
Primary care staff perceptions for continuation/discontinuation				
Unintended consequences of RESTORE				
